# Musculoskeletal pain in an ageing population: a cross-sectional analysis of the Maastricht study

**DOI:** 10.1007/s00296-025-05961-w

**Published:** 2025-08-19

**Authors:** Saskia P.M. Truijen, Annelies Boonen, Carla J.H. van der Kallen, Annemarie Koster, Marloes van Onna

**Affiliations:** 1 Department of Rheumatology, Maastricht University Medical Centre+, P. Debyelaan 25, Maastricht, 6229 HX The Netherlands; 2 Care and Public Health Research Institute (CAPHRI), Maastricht University, Maastricht, The Netherlands; 3 Cardiovascular Research Institute Maastricht (CARIM), Maastricht University, Maastricht, The Netherlands; 4 Department of Internal Medicine, Maastricht University Medical Centre+, Maastricht, The Netherlands; 5 Department of Social Medicine, Maastricht University, Maastricht, The Netherlands

**Keywords:** Musculoskeletal diseases, Chronic pain, Age groups, Epidemiology, Musculoskeletal pain

## Abstract

To estimate the prevalence of musculoskeletal pain (MSP) across age groups in a Dutch population sample of middle-aged and older adults. Cross-sectional data of 8618 participants aged 40–75 years from The Maastricht Study was used. Participants reported any (≥ 1 day in the past month) and chronic (≥ 3 months) MSP. Additionally, the latter group reported pain locations (grouped in six pain regions) and intensity (visual analogue scale 1–10). Prevalence of any and (patterns of) chronic MSP were described across seven age groups. Logistic and multinominal regression analyses assessed associations of age groups with MSP, adjusted for sex or type 2 diabetes mellitus (T2DM). Interactions between age and sex or T2DM were tested. Prevalence of any and chronic MSP was 53% and 29%, respectively. Any MSP was less common in participants aged 70–75 years compared to those aged 40–44 years (OR = 0.80, 95% CI: 0.65–0.99). Chronic MSP plateaued after age 60 (range ORs 55–69 years 1.27–1.38). Neck/chest/shoulder (65%) and lower back (55%) were the most common chronic pain regions. Pain in four or more locations was reported by 43%, with no age-group differences. Sex and T2DM did not modify the relation between age group and chronic MSP. Mean pain intensity in those with chronic MSP (5.6, SD 1.7) was stable across age groups. The prevalence of chronic MSP seems not to increase further after the age of 60, possibly reflecting decreased work demands, altered pain perception, effective pain management, or selection bias of healthier older individuals.

**Supplementary Information:**

The online version contains supplementary material available at 10.1007/s00296-025-05961-w.

## Introduction

The prevalence of chronic pain in the European general population ranges from 12 to 30%, with middle-aged adults appearing to be more likely than others to suffer from chronic pain [1]. One of the most common types of chronic pain is musculoskeletal pain (MSP) [2]. MSP is associated with substantial functional limitations and psychological distress, negatively impacting individuals’ health-related quality of life (HRQoL) [3], and increasing healthcare utilization [4–6]. 

Factors associated with chronic MSP include being female, a lower socio-economic position, past traumatic injury [7], occupational factors such as performing repetitive tasks or high job demands [8], lifestyle factors such as smoking and alcohol consumption, and presence of rheumatic and musculoskeletal disease (RMD) [9, 10]. Furthermore, advancing age has been reported as a risk factor of chronic MSP [11]. In a cross-sectional study in the UK, three out of four adults aged 45 years or older reported joint pain [12]. An increase in chronic pain when persons age might be explained by a combination of factors, such as the onset of osteoarthritis (OA), sarcopenia, and a higher incidence of conditions such as fragility fractures due to osteoporosis [11, 13, 14]. Moreover, with increasing age come increasing levels of multimorbidity, which is often accompanied by MSP [15, 16]. 

Previous research has shown conflicting results regarding the course of chronic MSP with age. Various studies show the prevalence of chronic MSP increases up to 65 years of age, with a plateau or decline thereafter [5, 17]. This was explained by a decreasing physical and mental burden from (paid) work participation after the age of retirement [5]. In contrast, another study showed a progressive increase of chronic MSP with increasing age in women, whereas in men the prevalence only increased after the age of 75 years [18]. Few studies address ‘any’ MSP, regardless of chronicity. More insight into this broader perspective is important, as even temporary or intermittent MSP can result in a substantial impact on HRQoL and the need for (informal) care and healthcare. In the context of population ageing, insight into the knowledge gaps regarding MSP in older persons – as well as the prevalence and patterns of chronic MSP – are of importance to understand the health burden of ageing and gain insight into healthcare planning.

The Maastricht Study is an observational, prospective, population-based cohort study among individuals between the ages of 40–75 years old, in which data are collected on the prevalence and patterns (i.e. pain regions, pain intensity, number of painful locations) of any and chronic MSP in the Netherlands. This study offers the opportunity to gain insight into the prevalence of MSP and pain patterns in a general population and how this prevalence differs in different age groups. The objective of the current study was therefore to estimate the prevalence of any and (patterns of) chronic MSP across middle-aged and older age groups.

## Materials and methods

### Study design and population

Data were used from The Maastricht Study, an observational prospective population-based cohort study. The rationale and methodology of this study have been described previously [19]. In brief, the study focuses on the etiology, pathophysiology, complications, and comorbidities of T2DM and is characterized by an extensive phenotyping approach. Eligible for participation were all individuals aged between 40 and 75 years living in the southern part of the Netherlands. Participants were recruited through mass media campaigns, from the municipal registries, and the regional Diabetes Patient Registry via mailings. Recruitment was stratified according to known T2DM status, with oversampling of individuals with T2DM.

The present study includes cross-sectional data from the first 9187 participants, who were included between November 2010 and October 2020. The examinations of each participant were performed within a time window of three months. Individuals aged below 40 years or above 75 years, and those who had not completed the pain questionnaire, were excluded. The study has been approved by the institutional medical ethical committee of the Academic Hospital Maastricht/Maastricht University (NL31329.068.10) and the Ministry of Health, Welfare and Sports of the Netherlands (Permit 131088-105234-PG). All participants gave written informed consent.

### Musculoskeletal pain

The presence of MSP was assessed by a self-reported questionnaire based on the National Health and Nutrition Survey (NHANES) pain questionnaire [20]. Participants were asked whether they experienced joint, neck, back and/or chest pain (yes/no) on at least one day in the past month (further called ‘any MSP’). If any MSP was or had been present, participants were asked whether the pain was still present, and if yes, whether the pain lasted at least three months (further called ‘chronic MSP’ [4]). Persons with (any or chronic) MSP were asked to indicate MSP locations (neck, low back, shoulder, elbow, wrist, hand, pelvis, hip, knee, ankle, foot, chest or everywhere) and to indicate the most painful location. Participants with chronic MSP were asked whether the pain was a result of a traumatic injury (yes/no), whether a general practitioner (GP) or medical specialist had provided a rheumatological diagnosis for the MSP (yes/no) and if so which RMD(s) had been diagnosed (tendinitis or bursitis, osteoporosis, fibromyalgia, chronic widespread pain, hypermobility, OA, chronic back pain, rheumatoid arthritis, psoriatic arthritis, axial spondyloarthritis, reactive arthritis, systemic lupus erythematosus (SLE), gout, or another rheumatologic disorder). Finally, participants with chronic MSP indicated pain intensity on a VAS scale from 1 (very little pain) to 10 (unbearable pain). To facilitate analyses by MSP locations, the 13 MSP locations were grouped into six anatomical regions: (1) pain everywhere, (2) knee, ankle and/or foot, (3) chest, neck and/or shoulder, (4) low back, (5) elbow, wrist and/or hand, and (6) pelvis and/or hip. Additionally, based on the 13 MSP locations, the total number of MSP locations was grouped into one, two, three or four or more MSP locations.

### Covariates

In The Maastricht Study, a broad range of variables have been collected through questionnaires, physical examination, biobanking and imaging techniques. For the current study, participants’ demographic characteristics included age (years), sex (male or female), educational level (low (no education, primary education, or lower vocational education), middle (intermediate vocational education or higher secondary education), high (higher vocational education or university education)), living situation (independent dwelling, retirement home/care home/sheltered housing, nursing home, other), smoking status (never, former, current) and alcohol consumption (none, low (women: ≤7 glasses/week, men: ≤14 glasses/week), high (women: >7 glasses/week, men: >14 glasses/week)). Body Mass Index (BMI) was calculated from measurements of height and weight. Glucose metabolism status was defined according to the World Health Organization 2006 criteria into normal glucose metabolism, prediabetes, T2DM, and other types of diabetes [19]. For the purposes of this study, participants were categorized as having T2DM or no T2DM (normal glucose metabolism, prediabetes and other types of diabetes).

### Statistical analysis

Sociodemographic characteristics, lifestyle characteristics and T2DM status of participants were described for the total population and stratified for seven five-year age groups using descriptive statistics. Continuous variables were presented as mean (SD) or median (IQR) for normally and non-normally distributed variables, respectively. Categorical variables were presented as percentages. To test differences across age groups, ANOVA and chi-square tests were used for continuous and categorical variables, respectively. To account for multiple testing in the age group comparisons involving MSP regions, a Bonferroni correction was applied by multiplying each *p*-value by the number of comparisons (*n* = 6).

Prevalence of any and chronic MSP were calculated for the total population and stratified by age groups. Additionally, for the chronic MSP population, prevalence of MSP regions and the number of painful locations (grouped in categories) were calculated. To assess whether the oversampling of participants with T2DM influenced prevalence estimates, prevalences of any and chronic MSP were directly standardized to the true sex- and age-specific T2DM prevalence in the Dutch population [21]. 

To assess the association of age groups with any MSP, chronic MSP and each of the six chronic MSP regions, uni- and multivariable logistic regression analyses were applied (effect size odds ratio (OR)), and multinomial regressions (effect size relative risk ratio (RRR)) when the number of chronic MSP locations was the outcome, with the age category 40–44 years as reference group (model 1). Cubic splines with five age knots were created to visualize the association of age with MSP patterns, with additional adjustment for T2DM when visualizing the prevalence of any or chronic MSP [22]. 

To understand whether sex and T2DM status confounded the association of age and MSP patterns or had an independent influence on MSP, regressions were repeated adjusting for sex (model 2) and T2DM (model 3). To assess whether sex and T2DM modified the association of age with MSP patterns, interactions with sex and T2DM status (age groups*sex and age groups*T2DM status) were included in model 2 and 3, respectively.

Prevalence of RMD diagnoses and the mean pain intensity were calculated for the chronic MSP population and stratified by age groups.

Statistical analyses were performed using STATA (StataCorp version 17). A *p*-value of < 0.05 was considered statistically significant.

## Results

### Study population characteristics

Of the 9187 participants enrolled in The Maastricht Study, 8618 participants (mean age 59.3 years, 50% women, 98% living independently) completed the pain questionnaire and were included for analysis (Supplementary Fig. 1). Overall, 40% had a high and 31% a low educational level, 13% were smokers, 59% were low alcohol consumers, the median BMI was 26.2 and 21% of participants had T2DM (Table 1). The youngest age group of 40–44 years old had a higher percentage of individuals with high education levels compared to those aged 65–75 years old with a higher percentage of individuals with lower education levels. The older age groups of 60–75 years seemed to be more often former but not current smokers, reported higher alcohol consumption, and had a higher median BMI, although trends were not statistically tested.

**Table 1:** Characteristics of the total study population by 5-year age intervals (*n* = 8618)

	Total	40–44 yrs	45–49 yrs	50–54 yrs	55–59 yrs	60–64 yrs	65–69 yrs	70–75 yrs	*p*-value
Number of participants, n (%)	8618 (100.0)	526 (6.1)	865 (10.0)	1172 (13.6)	1524 (17.7)	1748 (20.3)	1754 (20.4)	1029 (11.9)	-
Age (years), mean (SD)	59.3 (8.7)	42.2 (1.4)	47.1 (1.4)	52.1 (1.4)	57.1 (1.4)	62.1 (1.4)	66.8 (1.4)	72.0 (1.6)	-
Sex, n women (%)	4347 (50.4)	317 (60.3)	497 (57.5)	662 (56.5)	827 (54.3)	851 (48.7)	783 (44.6)	410 (39.8)	< 0.01*
*Educational level, n (%)*									< 0.01*
Low	2670 (31.0)	59 (11.2)	131 (15.1)	276 (23.6)	392 (25.7)	610 (34.9)	731 (41.7)	471 (45.8)	
Middle	2380 (27.6)	183 (34.8)	352 (40.7)	403 (34.4)	455 (29.9)	397 (22.7)	362 (20.6)	228 (22.2)	
High	3448 (40.0)	282 (53.6)	379 (43.8)	478 (40.8)	666 (43.7)	710 (40.6)	634 (36.2)	299 (29.1)	
Unknown	120 (1.4)	2 (0.4)	3 (0.4)	15 (1.3)	11 (0.7)	31 (1.8)	27 (1.5)	31 (3.0)	
*Living situation, n (%)*									0.18
Independent dwelling	8418 (97.7)	517 (98.3)	846 (97.8)	1142 (97.4)	1490 (97.8)	1710 (97.8)	1712 (97.6)	1001 (97.3)	
Retirement or care home/sheltered housing	18 (0.2)	1 (0.2)	1 (0.1)	0 (0.0)	3 (0.2)	2 (0.1)	5 (0.3)	6 (0.6)	
Other housing situation	113 (1.3)	7 (1.3)	15 (1.7)	15 (1.3)	23 (1.5)	22 (1.3)	21 (1.2)	10 (1.0)	
Unknown	69 (0.8)	1 (0.2)	3 (0.4)	15 (1.3)	8 (0.5)	14 (0.8)	16 (0.9)	12 (1.8)	
*Smoking status, n (%)*									< 0.01*
Never	3328 (38.6)	285 (54.2)	462 (53.4)	535 (45.7)	567 (37.2)	544 (31.1)	579 (33.0)	356 (34.6)	
Former	4127 (47.9)	157 (29.9)	278 (32.1)	424 (36.2)	731 (48.0)	963 (55.1)	988 (56.3)	586 (57.0)	
Current	1094 (12.7)	83 (15.8)	122 (14.1)	200 (17.1)	216 (14.2)	226 (12.9)	174 (9.9)	73 (7.1)	
Unknown	69 (0.8)	1 (0.2)	3 (0.4)	13 (1.1)	10 (0.7)	15 (0.9)	13 (0.7)	14 (1.4)	
BMI, median (IQR)	26.2 (23.7–29.1)	24.9 (22.5–28.1)	25.7 (23.3–29.0)	25.7 (23.1–28.7)	26.1 (23.7–29.1)	26.3 (23.9–29.2)	26.4 (24.1–29.4)	26.9 (24.4–29.9)	< 0.01*
*Alcohol consumption, n (%)*									< 0.01*
None	1514 (17.6)	97 (18.4)	176 (20.4)	231 (19.7)	252 (16.5)	277 (15.9)	285 (16.3)	196 (19.1)	
Low	5099 (59.2)	371 (70.5)	555 (64.2)	713 (60.8)	917 (60.2)	994 (56.9)	979 (55.8)	570 (55.4)	
High	1933 (22.4)	57 (10.8)	131 (15.1)	214 (18.3)	345 (22.6)	462 (26.4)	477 (27.2)	247 (24.0)	
Unknown	72 (0.8)	1 (0.2)	3 (0.4)	14 (1.2)	10 (0.7)	15 (0.9)	13 (0.7)	16 (1.6)	
T2DM, n (%)	1776 (20.6)	32 (6.1)	88 (10.2)	154 (13.1)	269 (17.7)	387 (22.1)	479 (27.3)	367 (35.7)	< 0.01*

*BMI* Body Mass Index, *T2DM* Type 2 Diabetes Mellitus

*Chi^2^ or ANOVA test *p*-value < 0.05

### Any MSP

**Fig. 1:**
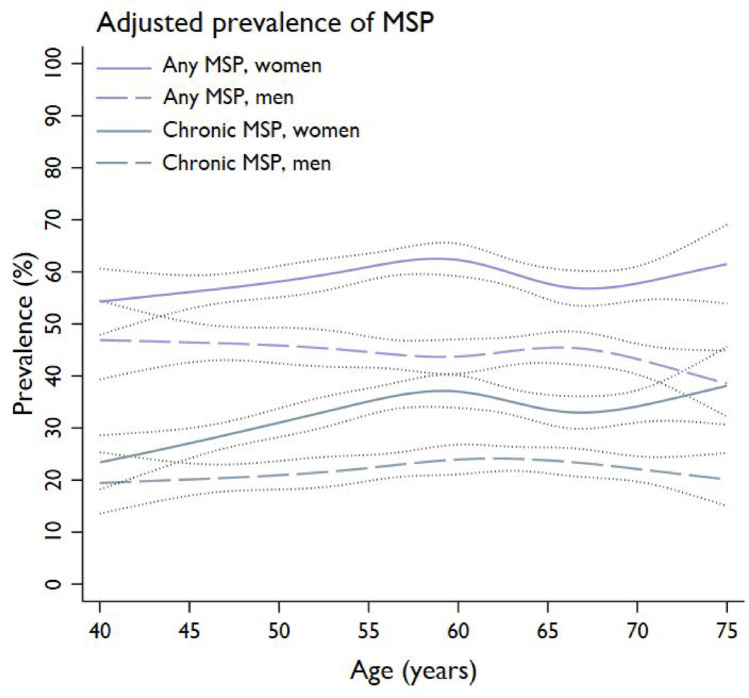
Prevalence (95% CI) of any and chronic MSP by age in the total population, adjusted for T2DM and stratified for men and women (*n* = 8618)

The prevalence of any MSP was 53% (*n* = 4541/8618). Although a lower likelihood on any MSP was observed in those aged 70–75 years compared to the youngest age group of 40–44 years (48% vs. 53%; OR = 0.80, 95% CI: 0.65–0.99) (Table 2 and Supplementary Fig. 2), this association became non-significant after adjusting for sex (Table 3 (model 1 and 2)). No association of other age groups with any MSP were found. Overall, women (60%) were more likely to experience any MSP compared to men (45%) (OR_women_=1.82, 95% CI: 1.67–1.99) (Table 3 (model 2) and Supplementary Table 1). No association between the presence of T2DM and any MSP was found (crude prevalence 53% in both groups; OR_T2DM_=1.06, 95% CI: 0.95–1.18) (Table 3 (model 3) and Supplementary Fig. 3a). Crude and T2DM-standardized prevalences were comparable in the total population (52.7% vs. 52.3%, respectively), and in the seven age groups (Supplementary Table 1). Sex and T2DM did not modify the associations of age groups with any MSP (interactions *p* > 0.05). Figure 1 presents the prevalence of any MSP by age, stratified by sex and adjusted for T2DM.

**Table 2:** Prevalence of any and chronic MSP in the total population (*n* = 8618), and MSP regions, RMD diagnoses and pain characteristics in the chronic MSP population by 5-year age intervals (*n* = 2513)

	Total	40–44 yrs	45–49 yrs	50–54 yrs	55–59 yrs	60–64 yrs	65–69 yrs	70–75 yrs	*p*-value
Number of participants, n (%)	8618 (100.0)	526 (6.1)	865 (10.0)	1172 (13.6)	1524 (17.7)	1748 (20.3)	1754 (20.4)	1029 (11.9)	-
Any MSP, n (%)	4541 (52.7)	280 (53.2)	447 (51.7)	633 (54.0)	832 (54.6)	933 (53.4)	924 (52.7)	492 (47.8)	0.03
Chronic MSP, n (%)	2513 (29.2)	132 (25.1)	207 (24.0)	339 (29.0)	475 (31.2)	551 (31.6)	523 (29.8)	286 (27.8)	< 0.01*
Chronic MSP population (*n* = 2513)									
*Pain regions, n (%)* ^a^									
Everywhere	170 (6.8)	3 (2.3)	15 (7.3)	30 (8.9)	37 (7.8)	27 (4.9)	33 (6.3)	25 (8.7)	0.32
Knee, ankle and/or foot	1311 (52.2)	51 (38.6)	100 (48.3)	161 (47.5)	266 (56.0)	315 (57.2)	278 (53.2)	140 (49.0)	< 0.01**
Neck, chest and/or shoulder	1638 (65.2)	92 (69.7)	141 (68.1)	236 (69.6)	326 (68.6)	337 (61.2)	334 (63.9)	172 (60.1)	0.13
Low back	1383 (55.0)	86 (65.2)	116 (56.0)	188 (55.5)	256 (53.9)	304 (55.2)	278 (53.2)	155 (54.2)	1.00
Elbow, wrist and/or hand	1013 (40.3)	39 (29.6)	80 (38.7)	136 (40.1)	210 (44.2)	234 (42.5)	222 (42.5)	92 (32.2)	0.02**
Hip and/or pelvis	809 (32.2)	46 (34.9)	60 (29.0)	102 (30.1)	159 (33.5)	185 (33.6)	166 (31.7)	91 (31.8)	1.00
*Number of MSP locations, n (%)*									0.03*
1	374 (14.9)	19 (14.4)	31 (15.0)	51 (15.0)	56 (11.8)	75 (13.6)	85 (16.3)	57 (19.9)	
2	554 (22.1)	32 (24.2)	56 (27.1)	61 (18.0)	95 (20.0)	133 (24.1)	117 (22.4)	60 (21.0)	
3	497 (19.8)	32 (24.2)	37 (17.9)	56 (16.5)	103 (21.7)	101 (18.3)	112 (21.4)	56 (19.6)	
≥ 4	1088 (43.3)	49 (37.1)	83 (40.1)	171 (50.4)	221 (46.5)	242 (43.9)	209 (40.0)	113 (39.5)	
*RMD diagnosis , n (%)* ^b, c^									< 0.01*
Yes	734 (29.2)	21 (15.9)	50 (19.9)	90 (26.5)	130 (27.2)	170 (30.9)	177 (33.8)	96 (33.6)	
Osteoarthritis	203 (8.1)	3 (2.3)	11 (5.3)	18 (5.3)	39 (8.2)	43 (7.8)	65 (12.4)	24 (8.4)	
Fibromyalgia	125 (5.0)	5 (3.8)	16 (7.7)	23 (6.8)	28 (5.9)	29 (5.3)	17 (3.3)	7 (2.5)	
Tendinitis or bursitis	61 (2.4)	1 (0.8)	2 (1.0)	11 (3.2)	7 (1.5)	19 (3.5)	16 (3.1)	5 (1.8)	
Other	345 (13.7)	12 (9.1)	21 (10.1)	38 (11.2)	56 (11.8)	79 (14.3)	79 (15.1)	60 (21.0)	
No	1777 (70.7)	111 (84.1)	157 (75.9)	249 (73.5)	344 (72.4)	381 (69.2)	346 (66.2)	189 (66.1)	
Pain by trauma, n (%) ^d^	295 (11.8)	21 (15.9)	26 (12.6)	37 (10.9)	53 (11.2)	62 (11.3)	58 (11.1)	38 (13.3)	0.72
Pain intensity, mean (SD) ^d^	5.6 (1.7)	5.8 (1.6)	5.5 (1.7)	5.7 (1.8)	5.6 (1.7)	5.5 (1.7)	5.6 (1.8)	5.8 (1.7)	0.47

*MSP* Musculoskeletal Pain, *RMD* Rheumatic and Musculoskeletal Diseases

*Chi^2^ or ANOVA test *p*-value < 0.05

**p-value < 0.05 after Bonferroni correction

^a^Bonferroni correction was applied for age group comparisons of pain regions

^b^Only participants with MSP completed the RMD questionnaire

^c^No. of participants with a missing value *n* = 2

^d^Based on 2511/2513 participants

**Table 3:** Univariable and multivariable logistic regression analyses of the association between age groups and any and chronic MSP (*n* = 8618)

Any MSP	Model 1		Model 2		Model 3	
	OR (95% CI)	*p*-value	OR (95% CI)	*p*-value	OR (95% CI)	*p*-value
*Age group, years*						
40–44	Reference	-	Reference	-	Reference	-
45–49	0.94 (0.76–1.17)	0.57	0.95 (0.77–1.19)	0.68	0.94 (0.75–1.16)	0.56
50–54	1.03 (0.84–1.27)	0.77	1.06 (0.86–1.30)	0.61	1.03 (0.84–1.26)	0.80
55–59	1.06 (0.87–1.29)	0.59	1.10 (0.90–1.34)	0.37	1.05 (0.86–1.28)	0.64
60–64	1.01 (0.83–1.22)	0.95	1.08 (0.89–1.32)	0.45	1.00 (0.82–1.21)	0.97
65–69	0.98 (0.80–1.19)	0.82	1.08 (0.88–1.31)	0.47	0.97 (0.79–1.18)	0.73
70–75	0.80 (0.65–0.99)	0.04*	0.91 (0.73–1.12)	0.37	0.79 (0.64–0.98)	0.03*
Sex, *female*	-	-	1.82 (1.67–1.99)	< 0.01*	-	-
T2DM, yes	-	-	-	-	1.06 (0.95–1.18)	0.27

Chronic MSP^a^	Model 1		Model 2		Model 3	
	OR (95% CI)	*p*-value	OR (95% CI)	*p*-value	OR (95% CI)	*p*-value
*Age group, years*						
40–44	Reference	-	Reference	-	Reference	-
45–49	0.94 (0.73–1.21)	0.62	0.95 (0.74–1.23)	0.71	0.93 (0.72–1.20)	0.58
50–54	1.22 (0.96–1.54)	0.10	1.25 (0.98–1.58)	0.07	1.20 (0.95–1.52)	0.13
55–59	1.35 (1.08–1.70)	0.01*	1.41 (1.12–1.77)	< 0.01*	1.33 (1.06–1.66)	0.01*
60–64	1.38 (1.10–1.72)	0.01*	1.48 (1.18–1.85)	< 0.01*	1.34 (1.07–1.67)	0.01*
65–69	1.27 (1.02–1.58)	0.04*	1.39 (1.11–1.74)	< 0.01*	1.22 (0.98–1.53)	0.08
70–75	1.15 (0.91–1.46)	0.25	1.29 (1.02–1.65)	0.04*	1.09 (0.86–1.39)	0.49
Sex, female	-		1.76 (1.60–1.94)	< 0.01*	-	-
T2DM, yes	-		-	-	1.20 (1.07–1.34)	< 0.01*

*MSP* Musculoskeletal Pain, *T2DM* Type 2 Diabetes Mellitus

*p-value < 0.05

^a^Based on 8612/8618 participants

### Chronic MSP

The prevalence of chronic MSP was 29% (*n* = 2513/8618), varying from 25% in ages 40–44 to 32% in 60–64, and 28% in those aged 70–75 years (Table 2). Individuals aged 55–69 years were more likely to experience chronic MSP compared to the youngest age group of 40–44 years, with unadjusted ORs appearing to plateau from age 60 onwards (55–59 yrs: OR = 1.35, 95% CI: 1.08–1.70, 60–64 yrs: OR = 1.38, 95% CI: 1.10–1.72, 65–69 yrs: OR = 1.27, 95% CI: 1.02–1.58) (Tables 2 and 3 (model 1), and Supplementary Fig. 2). Chronic MSP was more prevalent in women (35%) compared to men (24%) (OR_women_=1.76, 95% CI: 1.60–1.94) (Table 3 (model 2) and Supplementary Table 1). After adjusting for sex, those aged 70–75 years were also more likely to experience chronic MSP (OR = 1.29, 95% CI: 1.02–1.65) (Table 3 (model 2)). Chronic MSP was more prevalent in those with T2DM (32%) compared to those without T2DM (28%) (OR_T2DM_=1.20, 95% CI: 1.07–1.34) (Table 3 (model 3) and Supplementary Fig. 3b). Notwithstanding, the T2DM-standardized prevalence of chronic MSP (28.6%) was comparable to the crude prevalence in the total group (29.2%) and in the seven age groups (Supplementary Table 1). Sex and T2DM did not modify the associations of age groups with chronic MSP (interactions *p* > 0.05). Figure 1 presents the prevalence of chronic MSP by age, stratified by sex and adjusted for T2DM.

### Chronic MSP regions

**Fig. 2:**
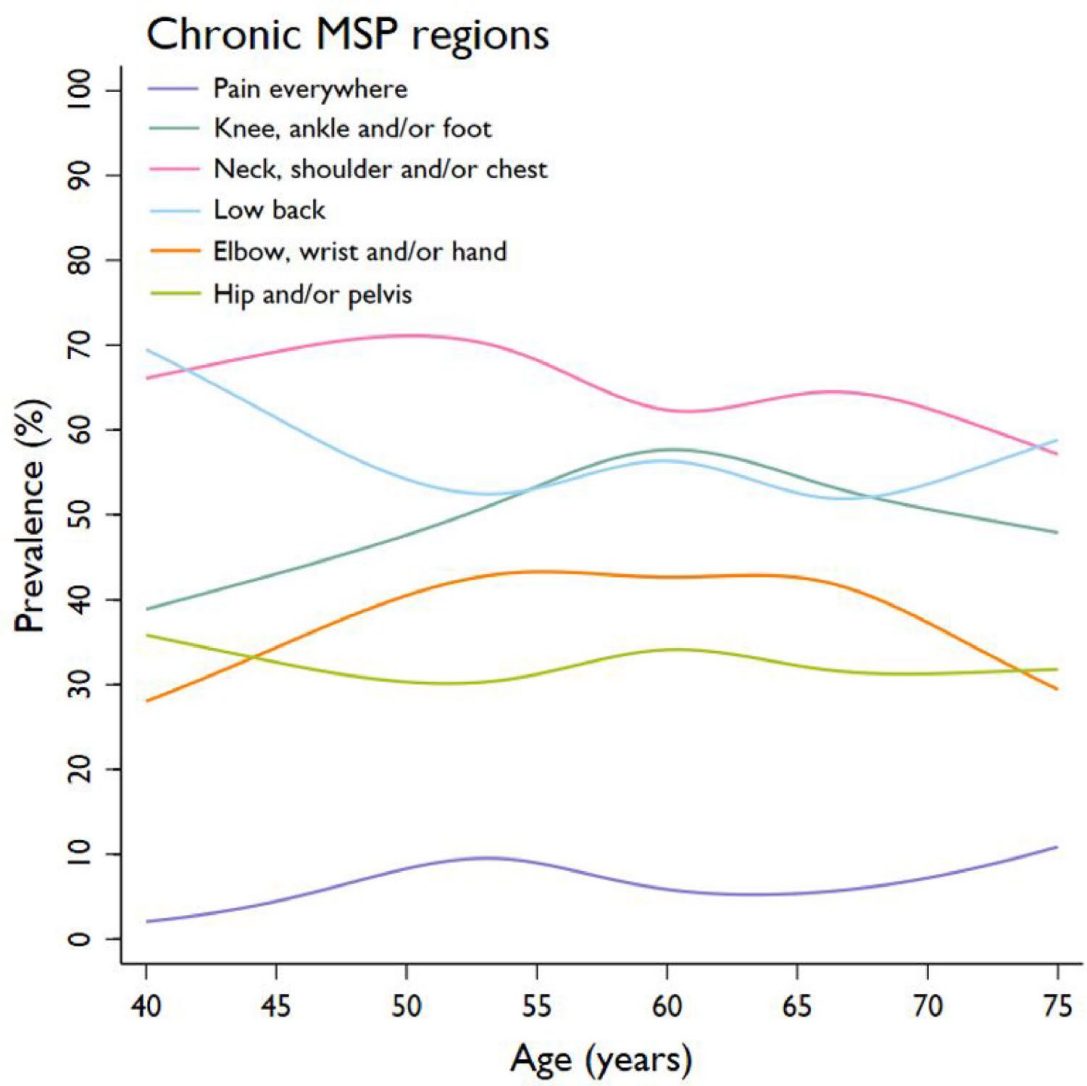
Prevalence of MSP regions by age in the chronic MSP population (*n* = 2513)

The most prevalent region of chronic MSP was the *neck*,* shoulder and/or chest* (65% (*n* = 1638/2513) in those with chronic MSP; 19% (*n* = 1638/8618) in the total population), followed by *low back pain* (55% (*n* = 1383/2513); 16% (*n* = 1383/8618)) and *knee*,* ankle and/or foot pain* (52% (*n* = 1311/2513); 15% (*n* = 1311/8618)) (Fig. 2; Table 2). The *neck*,* shoulder and/or ches*t was also most frequently reported as the most painful region (33% (*n* = 822/2513)). *MSP everywhere* was the least prevalent chronic MSP region (7% (*n* = 170/2513)), and reported by 2% (*n* = 170/8618) of the total population.

The adjusted likelihood of reporting *MSP everywhere* was statistically significantly higher for those aged 50–59 (8 to 9%; range OR 3.44–4.05) and 70–75 (9%; OR = 3.87) years compared to those aged 40–44 (2%) years (Table 2 and Supplementary Table 2). The likelihood of *low back pain* was lower among individuals aged 55–59 (54%; OR = 0.64) and 65–69 (53%; OR = 0.63) years compared to the youngest age group (65%). For *knee*,* ankle and/or foot* and *elbow*,* wrist and/or hand pain*, a higher prevalence was seen after the age of 40–44 years (from 39 to 57%; range OR 1.74–2.07 and from 30 to 44%; range OR 1.60–1.89, respectively), followed by a lower prevalence in the older age groups (from 57 to 49% and from 44 to 32%, respectively).

Women were systematically more likely than men to have MSP in each pain region (range OR 1.23–2.40), except for the lower back, where no difference between sexes was observed (Supplementary Tables 2 and Supplementary Fig. 4a-b). Individuals with T2DM were more likely to experience MSP everywhere and in the knee, ankle and/or foot (range OR 1.34–1.77). Sex and T2DM did not modify the association of age groups with chronic MSP regions (interactions *p* > 0.05), except one interaction age group*T2DM was found for neck, shoulder and/or chest pain, which was not considered relevant after stratification.

### Number of painful locations in chronic MSP

**Fig. 3:**
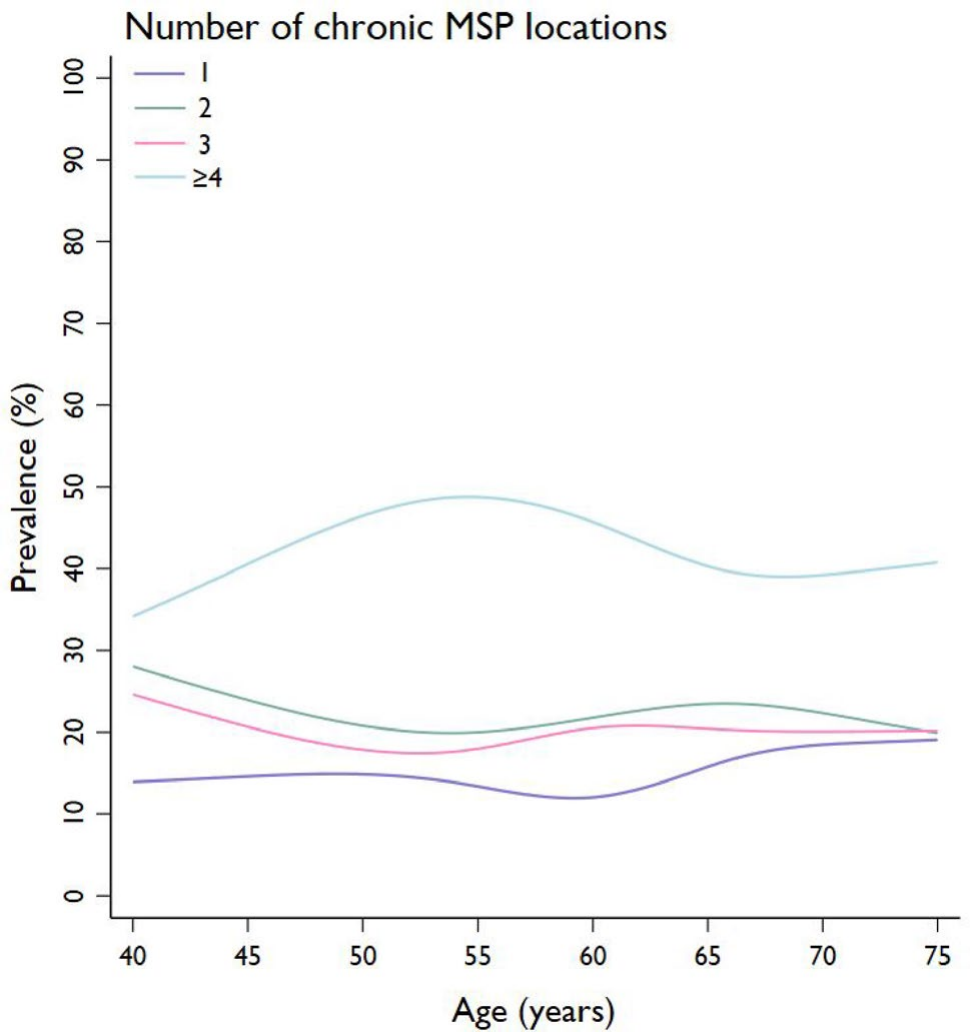
Prevalence of number of MSP locations by age in the chronic MSP population (*n* = 2513)

A total of 43% of the chronic MSP population (*n* = 1088/2513; 12.6% (*n* = 1088/8618) in the total population) reported pain at four or more MSP locations (Fig. 3; Table 2). Adjusted multinominal regression analysis showed no associations between age groups and the number of MSP locations (Supplementary Table 3). Women were more likely to report MSP in two, three and four or more MSP locations compared to men, and the likelihood seemed to increase numerically for each additional painful location (RRR_women_=1.40, 95% CI: 1.07–1.84; RRR_women_=1.95, 95% CI: 1.48–2.58; RRR_women_=3.49, 95% CI: 2.72–4.47, respectively). Those with T2DM were more likely to experience MSP in four or more MSP locations compared to those without T2DM (RRR_T2DM_=1.38, 95% CI: 1.02–1.85). Sex and T2DM did not modify the influence of age groups on the number of MSP locations (interactions *p* > 0.05).

### RMD diagnosis and pain intensity in chronic MSP

A total of 12% of those with chronic MSP reported their pain resulted from a trauma (*n* = 295/2513), while 29% (*n* = 734/2513) received a RMD diagnosis for chronic MSP, of which OA (8.1%, *n* = 203/2513; 28% of those with a RMD diagnosis), fibromyalgia (5.0%, *n* = 125/2513; 17% of those with a RMD diagnosis) and tendinitis or bursitis (2.4%, *n* = 61/2513; 8% of those with a RMD diagnosis) were most often reported (Table 2). The two oldest age groups reported most often a diagnosis of a RMD (65–69 yrs: 33.8% (*n* = 177/523) and 70–75 yrs: 33.6% (*n* = 96/286)). Mean pain intensity in those with chronic MSP was 5.6 (SD 1.7). Pain intensity appeared stable across age groups (Table 2), was comparable in women (5.8 (SD 1.7)) opposed to men (5.4 (SD 1.8)), but was higher when a RMD diagnosis was present (6.1 (SD 1.6) opposed to 5.4 (SD 1.7)).

## Discussion

This cross-sectional analysis showed that both any and chronic musculoskeletal pain (MSP) are common in individuals aged 40–75 years living in the south of the Netherlands, with prevalences of 53% and 29%, respectively. The neck, chest and shoulder region and lower back were the most commonly affected areas of chronic MSP. In crude analyses, the prevalence of any MSP did not differ across age groups. In contrast, the prevalence of chronic MSP increased until the age group of 60 and plateaued thereafter.

A previous Dutch population study among 3664 individuals aged 25 years and older reported a point prevalence of 54% for MSP at the time of the survey and 44% for chronic MSP [23]. The higher prevalence of chronic MSP in that study might be slightly overestimated due to selective non-response, as it was specifically designed to investigate MSP [23]. Furthermore, despite a similar definition considering the duration of chronic MSP, the questioning of the presence of MSP was slightly different, which could also explain discrepancies. A population study among 61,157 European adults aged 50 years or older estimated the prevalence of chronic MSP to be 36%, with a prevalence of 20% in the Netherlands [18]. The stricter criteria for defining chronic MSP (MSP in the past six months vs. three months in our study) may partly explain the lower prevalence of chronic MSP in that study.

Few studies have examined the impact of age on the epidemiology of pain, with conflicting findings. In our study, the chronic MSP prevalence was 25% among individuals aged 40–44 years and 32% in those aged 60–64 years, with similar prevalence levels in subsequent age groups. In contrast, the European study observed a significant progressive increase of chronic MSP by age in crude analysis, which disappeared in multivariable analysis [18]. In a Swedish population study, there was an increasing prevalence up to 55–64 years for men, and up to 65–69 years for women, followed by a decrease [24]. The common finding across these studies is the absence of a consistent progressive increase in chronic MSP prevalence with advancing age.

The absence of a consistently higher chronic MSP prevalence in older age groups is somewhat counterintuitive, as conditions like osteoarthritis and other RMDs are known to become more prevalent with age [25, 26]. The lack of an age-related increase in chronic MSP may partly be explained by the reduced physical and mental workload individuals experience after retirement [5]. Additionally, although findings on age-related changes in pain perception, including adjustments in pain sensitivity and thresholds, are conflicting, there may be indications that such alterations do occur [14, 27–29]. Furthermore, older individuals may also develop effective coping strategies over time. This enables them to manage chronic pain more effectively (e.g. by avoiding pain-inducing activities), potentially leading to fewer reports of new or worsening MSP as they age. Lastly, our findings may also reflect the effectiveness of current appropriate pain management strategies in adults over 60.

Alternative explanations may have a methodological basis. A potential factor could be selection bias, as older adults with considerable musculoskeletal complaints may refrain from participating in The Maastricht Study. On the other hand we might have missed the ‘tipping point’ for age, after which MSP prevalence starts to rise again. A previous study mentioned an age of 85 as tipping point for pain in the ageing process [30], while our population’s age range was 40 to 75 years. The prevalence of geriatric syndromes as frailty and sarcopenia have been shown to increase with age, especially in those aged 85 years or older [31]. 

Despite potential methodological limitations, several trends regarding age and the number of pain locations as well as the location of pain merit discussion. The majority of individuals with chronic MSP reported pain at four or more locations with a medium pain intensity. Pain in at least four locations peaked between ages 50–59 years. Consistent with previous research, the neck, chest and shoulder region and lower back were the most commonly affected areas of chronic MSP [16, 19, 28]. In our study, age was not associated with neck, chest, and/or shoulder pain, while low back pain appeared to occur less frequently in older age groups compared to the youngest group. The latter is in contrast to age-specific prevalence shown by the Global Burden of Disease (GBD) 2021 Study where a progressive increase in low back pain was observed until the age of 85 [32]. This discrepancy may be explained by the use of different definitions. In the GBD 2017 Study, low back pain was defined as pain lasting at least one day, thereby capturing both acute and chronic low back pain. In our study, only chronic low back pain (≥ 3 months) was investigated. Additionally, differences in population characteristics, healthcare systems and occupational exposures across countries may play a role [33]. For example, in the Netherlands, individuals in their early 50s may decide to refrain from heavy physical duties at work following consultation with their occupational physician, particularly as they approach retirement age.

Consistent with previous research [18, 23], women experienced pain more frequently any as well as chronic MSP in all pain regions, except for low back pain, where prevalence was not influenced by sex. However, the role of age in the different patterns of pain was similar between men and women. In women, the menopausal transition has been shown to be associated with joint pain [34], but this was not clearly confirmed in our study. Independent of age, T2DM was associated with chronic MSP. Since individuals with T2DM were oversampled in this cohort (20% compared to 6.0% in the Dutch population [21]), we standardized the prevalence of any and chronic MSP to the prevalence of T2DM in the Netherlands. This resulted in negligible differences, also within the individual age groups.

It was interesting to note that only 12% of those with chronic MSP reported their pain resulted from a trauma, and only 29% received a physician diagnosis, which was more prevalent in the older age groups. Older individuals may have been experiencing pain for a longer period, which could prompt them to seek medical help. Osteoarthritis followed by fibromyalgia were the most frequent diagnoses.

A strength of this study is the use of a large cohort of the general population in the south of the Netherlands. This focus may however limit the generalizability of our findings to other areas with differing sociodemographic and economic characteristics. Selection bias of individuals experiencing MSP is unlikely, as The Maastricht Study was designed to measure a wide range of outcomes rather than focusing specifically on MSP. In addition to the methodological limitations mentioned above, this study has several other limitations. First, the use of cross-sectional data limits our ability to capture the progression of MSP with age, as well as to establish a cause-and-effect relationship between ageing and MSP, as longitudinal data is currently unavailable. Second, the presence of a RMD diagnosis among those with chronic MSP was based on patient-reported physician-diagnosed disease. As a result, individuals without (chronic) MSP could not report an RMD diagnosis. Moreover, this self-report approach has not been validated within The Maastricht Study. Recall bias cannot be ruled out, and inaccuracies in the recalled diagnosis are possible. Third, we had no access to data on analgesics use. As a result, we were unable to determine whether reported pain intensity reflected untreated conditions or residual musculoskeletal symptoms. Last, we were not able to verify whether the NHANES pain questionnaire was formally validated.

Nevertheless, the current study offers a detailed view of MSP prevalence across middle-aged and older adults in a large, general population cohort. The age-stratified prevalence estimates of MSP patterns add to the limited body of evidence on this important public health topic. Future research should focus on longitudinal studies on MSP, including individuals over 75 years of age, in order to capture its progression with age. Given the absence of a higher MSP prevalence across older age groups, further insights into possible explanations, such as altered pain perception or coping strategies in older adults, would also be valuable.

In conclusion, this study highlights that both any and chronic MSP are highly prevalent in middle-aged and older adults. Notably, the globally increasing prevalence of RMDs does not appear to be accompanied by a corresponding higher prevalence of chronic MSP in older age groups. This may be attributed to factors as decreasing work demands, altered pain perception, effective coping strategies, improved pain management in older adults, or methodological factors such as selection bias of healthier older individuals.

## Supplementary Information

Below is the link to the electronic supplementary material.

Supplementary Material 1

## Data Availability

The data of this study derive from The Maastricht Study, but restrictions apply to the availability of these data, which were used under license for the current study. Data are, however, available from the authors upon reasonable request and with permission of The Maastricht Study management team.
