# Supplementary material for: Musculoskeletal pain in an ageing population: a cross-sectional analysis of the Maastricht study
Source: Rheumatol Int. 2025 Aug 19;45(9):200. doi: 10.1007/s00296-025-05961-w (PMC12364741; doi:10.1007/s00296-025-05961-w)
Supplement: Supplementary file 1 — Supplementary Material 1 [file 296_2025_5961_MOESM1_ESM.docx]

**Supplementary material**


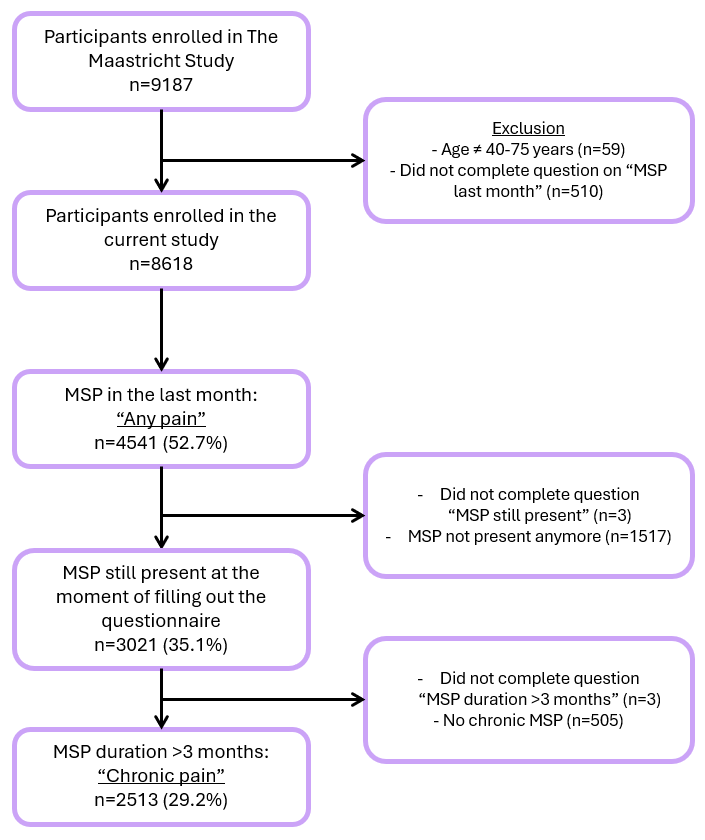


**Supplementary Fig 1.** Flowchart of the study population


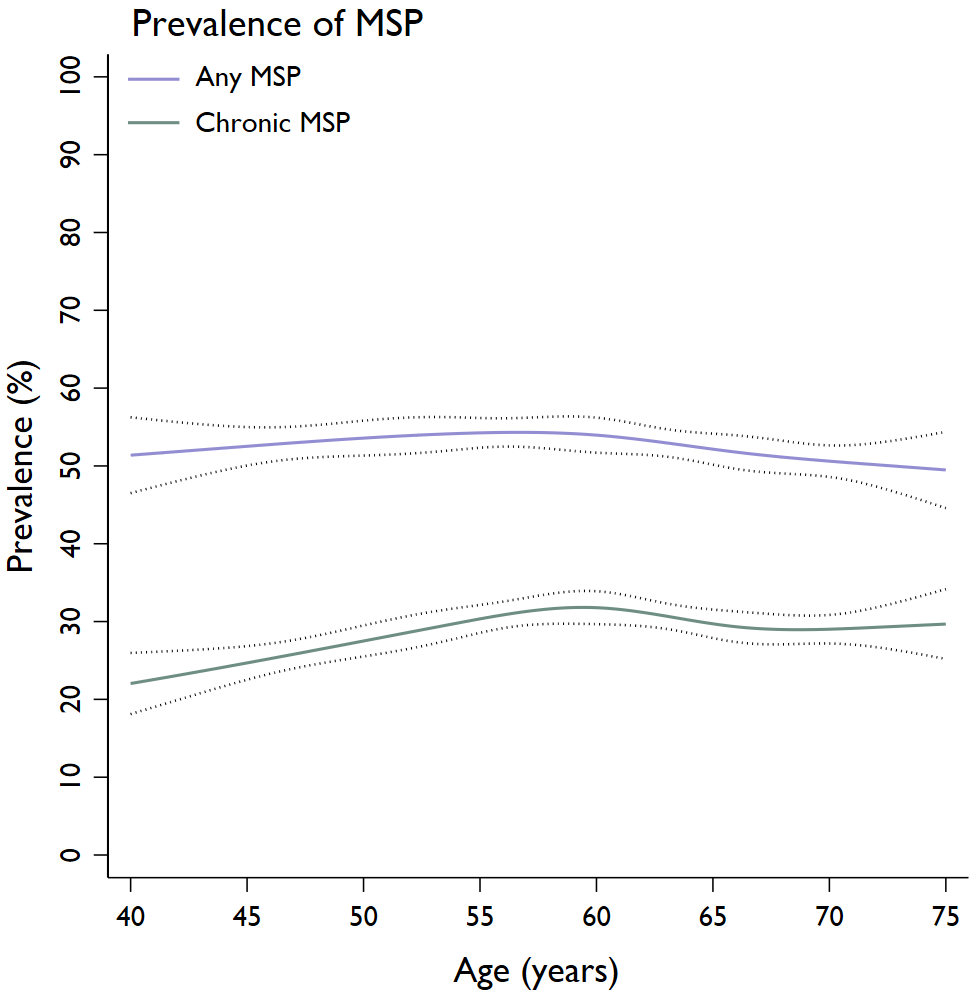


**Supplementary Fig 2.** Crude prevalence of any and chronic MSP in the total population (n=8618)


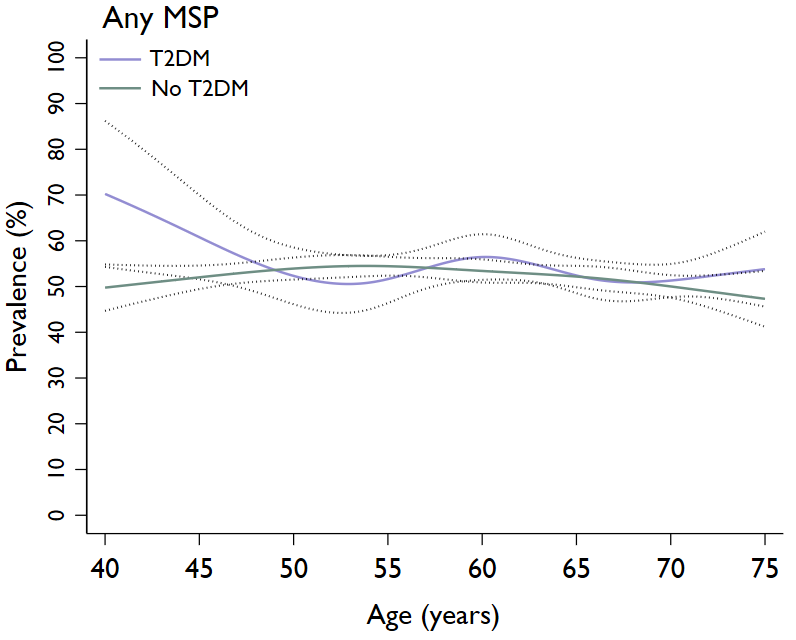


**Supplementary Fig 3a.** Prevalence of any MSP by T2DM status in the total population (n=8618)


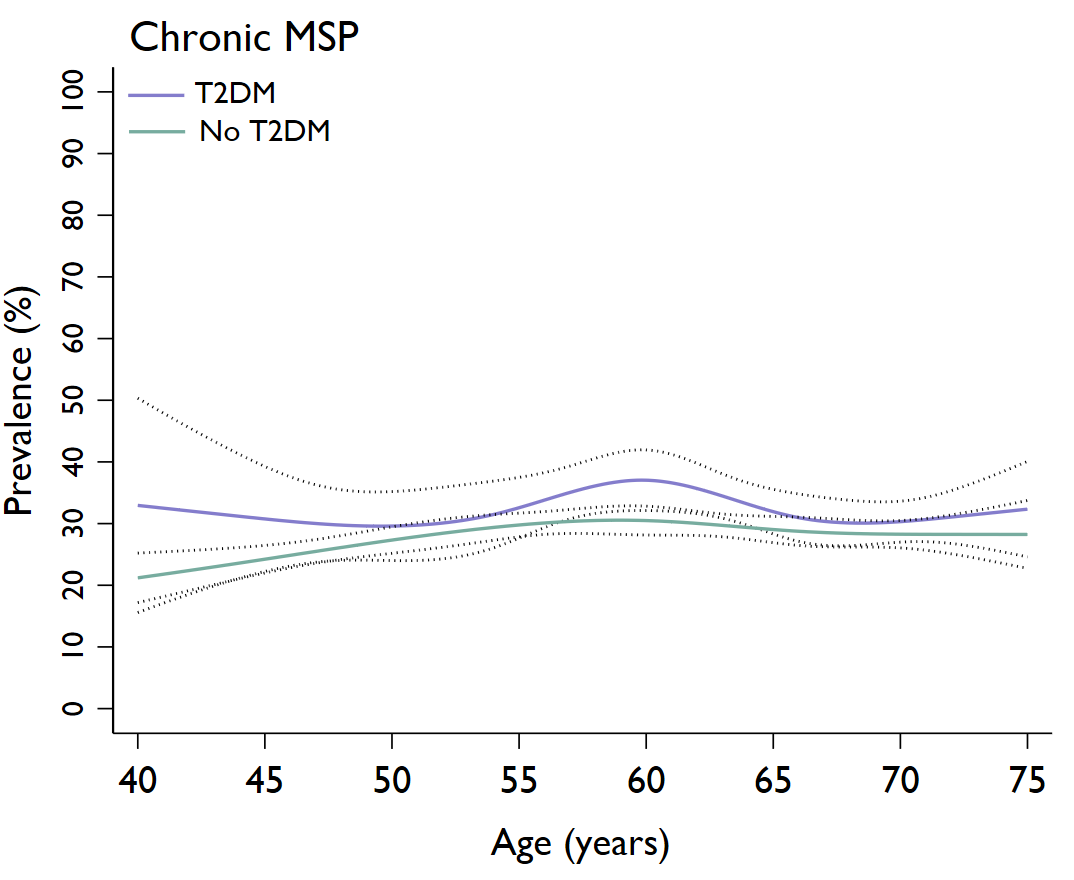


**Supplementary Fig 3b.** Prevalence of chronic MSP by T2DM status in the total population (n=8618)


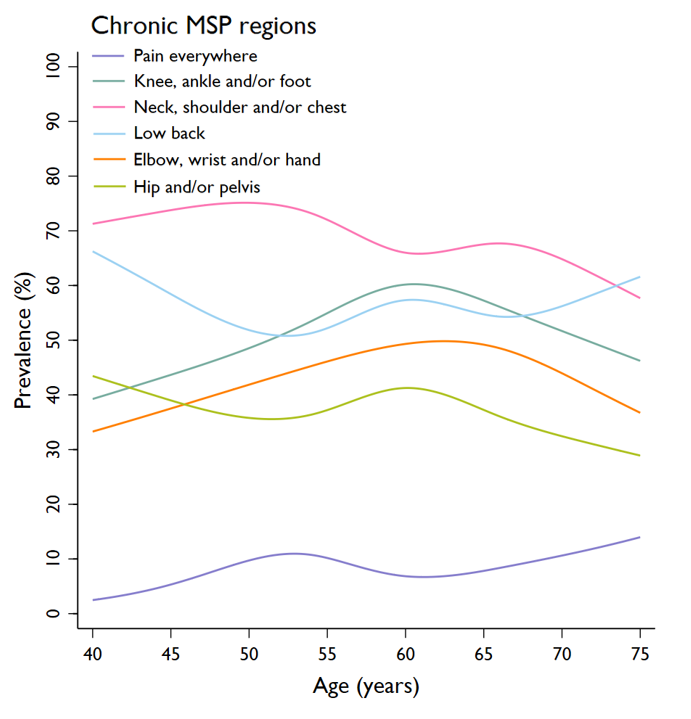


**Supplementary Fig 4a.** Prevalence of pain regions by age in the chronic MSP population (women) (n=1508)


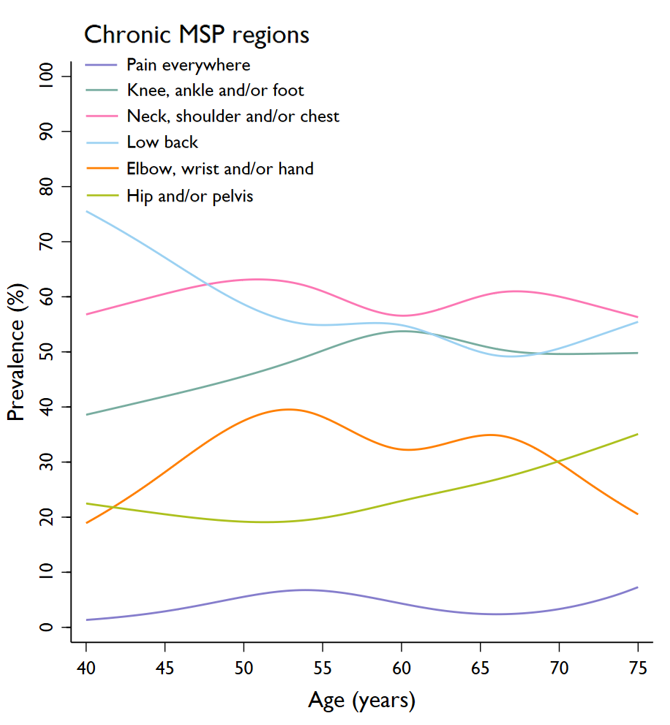


**Supplementary Fig 4b.** Prevalence of pain regions by age in the chronic MSP population (men) (n=1005)

| Supplementary Table 1. Crude and T2DM-adjusted prevalences of any and chronic MSP in the total population, stratified by sex and 5-year age intervals (n=8618) | | | | | | | | |
| --- | --- | --- | --- | --- | --- | --- | --- | --- |
|  | Total | 40-44 yrs | 45-49 yrs | 50-54 yrs | 55-59 yrs | 60-64 yrs | 65-69 yrs | 70-75 yrs |
| Number of participants, n (%) | 8618 (100.0) | 526 (6.1) | 865 (10.0) | 1172 (13.6) | 1524 (17.7) | 1748 (20.3) | 1754 (20.4) | 1029 (11.9) |
| **Any MSP** |  |  |  |  |  |  |  |  |
| Crude prevalence (%), total | 52.7 | 53.2 | 51.7 | 54.0 | 54.6 | 53.4 | 52.7 | 47.8 |
| T2DM-adjusted prevalence (%), total | 52.3 | 52.8 | 51.1 | 53.8 | 54.5 | 52.6 | 52.6 | 46.8 |
| Crude prevalence (%), men | 45.2 | 47.9 | 45.9 | 45.3 | 45.6 | 45.3 | 46.7 | 40.7 |
| T2DM-adjusted prevalence (%), men | 44.8 | 46.6 | 45.8 | 45.1 | 45.9 | 44.0 | 47.1 | 39.5 |
| Crude prevalence (%), women | 60.1 | 56.8 | 55.9 | 60.7 | 62.2 | 61.9 | 60.2 | 58.5 |
| T2DM-adjusted prevalence (%), women | 59.7 | 56.8 | 55.1 | 60.6 | 61.8 | 61.8 | 59.4 | 57.9 |
|  |  |  |  |  |  |  |  |  |
| **Chronic MSP** |  |  |  |  |  |  |  |  |
| Crude prevalence (%), total | 29.2 | 25.1 | 24.0 | 29.0 | 31.2 | 31.6 | 29.8 | 27.8 |
| T2DM-adjusted prevalence (%), total | 28.6 | 24.6 | 23.6 | 28.6 | 30.4 | 30.8 | 29.3 | 27.2 |
| Crude prevalence (%), men | 23.4 | 21.1 | 19.8 | 22.4 | 23.7 | 26.1 | 24.4 | 22.3 |
| T2DM-adjusted prevalence (%), men | 22.9 | 19.9 | 19.6 | 21.8 | 22.5 | 25.1 | 24.4 | 22.1 |
| Crude prevalence (%), women | 34.7 | 27.8 | 27.0 | 34.0 | 37.6 | 37.3 | 36.5 | 36.1 |
| T2DM-adjusted prevalence (%), women | 34.2 | 27.7 | 26.6 | 33.8 | 37.1 | 36.9 | 35.5 | 35.0 |
| **MSP:** Musculoskeletal Pain; **T2DM:** Type 2 Diabetes Mellitus | | | | | | | | |

| Supplementary Table 2. **Multivariable logistic regression analyses of the association between age groups and MSP regions in the chronic MSP population (n=2513)** | | | | | | |
| --- | --- | --- | --- | --- | --- | --- |
| **MSP regions**  ***OR (95% CI)*** ^a^ | Everywhere | Knee, ankle and/or foot | Neck, chest and/or shoulder | Low back | Elbow, wrist and/or hand | Hip and/or  pelvis |
| Age group, *years* |  |  |  |  |  |  |
| 40-44 | *reference* | *reference* | *reference* | *reference* | *reference* | *reference* |
| 45-49 | 3.33 (0.94–11.8) *p*=0.06 | 1.48 (0.95–2.31) *p*=0.09 | 0.94 (0.58–1.51) *p*=0.79 | 0.69 (0.44–1.08) *p*=0.10 | 1.52 (0.95–2.43) *p*=0.08 | 0.77 (0.48–1.23) *p*=0.27 |
| 50-54 | 4.05 (1.21–13.6) *p*=0.02* | 1.41 (0.94–2.13) *p*=0.10 | 1.00 (0.65–1.56) *p*=0.99 | 0.67 (0.44–1.02) *p*=0.06 | 1.60 (1.03–1.47) *p*=0.04* | 0.80 (0.52–1.23) *p*=0.31 |
| 55-59 | 3.44 (1.04–11.4) *p*=0.04* | 1.96 (1.32–2.92) *p*<0.01* | 0.97 (0.64–1.48) *p*=0.89 | 0.64 (0.43–0.95) *p*=0.03* | 1.89 (1.25–2.88)  *p*<0.01* | 0.94 (0.62–1.42) *p*=0.77 |
| 60-64 | 2.16 (0.64–7.25) *p*=0.21 | 2.07 (1.40–3.06)  *p*<0.01* | 0.72 (0.48–1.09) *p*=0.12 | 0.67 (0.45–1.00) *p*=0.05 | 1.83 (1.21–2.77)  *p*<0.01* | 0.99 (0.66–1.48) *p*=0.95 |
| 65-69 | 2.77 (0.83–9.24) *p*=0.10 | 1.74 (1.17–2.58)  *p*<0.01* | 0.82 (0.54–1.25) *p*=0.36 | 0.63 (0.42–0.93) *p*=0.02* | 1.84 (1.21–2.80)  *p*<0.01* | 0.92 (0.61–1.38) *p*=0.69 |
| 70-75 | 3.87 (1.14–13.2) *p*=0.03* | 1.44 (0.94–2.21) *p*=0.09 | 0.71 (0.46–1.12) *p*=0.14 | 0.66 (0.43–1.02)  *p*=0.06 | 1.18 (0.75–1.87) *p*=0.47 | 0.93 (0.60–1.46) *p*=0.77 |
| Sex, *female* | 2.40 (1.66–3.47)  *p*<0.01* | 1.23 (1.04–1.45) *p*=0.01* | 1.44 (1.22–1.71)  *p*<0.01* | 1.01 (0.86–1.19) *p*=0.87 | 1.73 (1.46–2.05)  *p*<0.01* | 1.86 (1.55–2.22)  *p*<0.01* |
| T2DM, *yes* | 1.77 (1.24–2.53)  *p*<0.01* | 1.34 (1.11–1.63)  *p*<0.01* | 0.89 (0.73–1.09)  *p*=0.25 | 0.87 (0.72–1.05)  *p*=0.15 | 1.13 (0.93–1.38) *p*=0.23 | 1.07 (0.87–1.32) *p*=0.52 |
|  |  |  |  |  |  |  |
| ^a^ Adjusted for sex and T2DM status; * *p*-value < 0.05  **MSP:** Musculoskeletal Pain; **T2DM:** Type 2 Diabetes Mellitus | | | | | | |

| Supplementary Table 3. **Multivariable multinominal logistic regression analysis of the association between age groups and the number of MSP locations in the chronic MSP population (n=2513)** | | | |
| --- | --- | --- | --- |
| **No. of MSP locations, *RRR (95% CI)* ^a^** | 2 vs. 1 | 3 vs. 1 | ≥4 vs. 1 |
| Age group, *years* |  |  |  |
| 40-44 | *reference* | *reference* | *reference* |
| 45-49 | 1.08 (0.53–2.22) *p*=0.83 | 0.72 (0.34–1.51)  *p*=0.38 | 1.05 (0.53–2.09)  *p*=0.88 |
| 50-54 | 0.72 (0.36–1.41)  *p*=0.33 | 0.66 (0.33–1.31)  *p*=0.23 | 1.31 (0.70–2.46)  *p*=0.39 |
| 55-59 | 1.02 (0.53–1.96)  *p*=0.97 | 1.10 (0.57–2.13) *p*=0.77 | 1.54 (0.83–2.85) *p*=0.17 |
| 60-64 | 1.09 (0.57–2.06) *p*=0.80 | 0.84 (0.44–1.61) *p*=0.61 | 1.35 (0.74–2.47) *p*=0.32 |
| 65-69 | 0.85 (0.45–1.61) *p*=0.62 | 0.83 (0.44–1.58) *p*=0.58 | 0.63 (0.31–1.24) *p*=0.18 |
| 70-75 | 0.65 (0.33–1.29) *p*=0.22 | 0.63 (0.31–1.24) *p*=0.18 | 0.85 (0.45–1.60) *p*=0.61 |
| Sex, *female* | 1.40 (1.07–1.84) *p*=0.01* | 1.95 (1.48–2.58)  *p*<0.01* | 3.49 (2.72–4.47)  *p*<0.01* |
| T2DM, *yes* | 1.04 (0.75–1.43) *p*=0.83 | 1.14 (0.82–1.58) *p*=0.45 | 1.38 (1.02–1.85) *p*=0.03* |
|  |  |  |  |
| ^a^ Adjusted for sex and T2DM status; * *p*-value < 0.05  **MSP:** Musculoskeletal Pain; **T2DM:** Type 2 Diabetes Mellitus | | | |
